# Predicting Later-Life Outcomes of Early-Life Exposures

**DOI:** 10.1289/ehp.1204934

**Published:** 2012-06-06

**Authors:** Kim Boekelheide, Bruce Blumberg, Robert E. Chapin, Ila Cote, Joseph H. Graziano, Amanda Janesick, Robert Lane, Karen Lillycrop, Leslie Myatt, J. Christopher States, Kristina A. Thayer, Michael P. Waalkes, John M. Rogers

**Affiliations:** 1 Department of Pathology and Laboratory Medicine, Division of Biology and Medicine, Brown University, Providence, Rhode Island, USA; 2 Departments of Developmental and Cell Biology and Pharmaceutical Sciences, University of California–Irvine, Irvine, California, USA; 3 Developmental and Reproductive Toxicology Center of Expertise, Drug Safety Research and Development, Pfizer Global Research and Development, Groton, Connecticut, USA; 4 Office of Research and Development, National Center for Environmental Assessment, U.S. Environmental Protection Agency, Washington, DC, USA; 5 Department of Environmental Health Sciences, Mailman School of Public Health, Columbia University, New York, New York, USA; 6 Developmental Origins of Health Laboratories, Division of Neonatology, Department of Pediatrics, University of Utah School of Medicine, Salt Lake City, Utah, USA; 7 Centre for Biological Sciences, Institute of Developmental Sciences, University of Southampton, Southampton, United Kingdom; 8 Department of Obstetrics and Gynecology, University of Texas Health Science Center, San Antonio, Texas, USA; 9 Department of Pharmacology and Toxicology, University of Louisville, Louisville, Kentucky, USA; 10 Division of the National Toxicology Program, National Institute of Environmental Health Sciences, National Institutes of Health, Department of Health and Human Services, Research Triangle Park, North Carolina, USA; 11 Toxicity Assessment Division, National Health Environmental Effects Research Laboratory, Office of Research and Development, U.S. Environmental Protection Agency, Research Triangle Park, North Carolina, USA

**Keywords:** arsenic, development, epigenetics, exposure, fetal, malnutrition, obesogen, PPAR

## Abstract

Background: *In utero* exposure of the fetus to a stressor can lead to disease in later life. Epigenetic mechanisms are likely mediators of later-life expression of early-life events.

Objectives: We examined the current state of understanding of later-life diseases resulting from early-life exposures in order to identify *in utero* and postnatal indicators of later-life diseases, develop an agenda for future research, and consider the risk assessment implications of this emerging knowledge.

Methods: This review was developed based on our participation in a National Research Council workshop titled “Use of *in Utero* and Postnatal Indicators to Predict Health Outcomes Later in Life: State of the Science and Research Recommendations.” We used a case study approach to highlight the later-life consequences of early-life malnutrition and arsenic exposure.

Discussion: The environmental sensitivity of the epigenome is viewed as an adaptive mechanism by which the developing organism adjusts its metabolic and homeostatic systems to suit the anticipated extrauterine environment. Inappropriate adaptation may produce a mismatch resulting in subsequent increased susceptibility to disease. A nutritional mismatch between the prenatal and postnatal environments, or early-life obesogen exposure, may explain at least some of the recent rapid increases in the rates of obesity, type 2 diabetes, and cardiovascular diseases. Early-life arsenic exposure is also associated with later-life diseases, including cardiovascular disease and cancer.

Conclusions: With mounting evidence connecting early-life exposures and later-life disease, new strategies are needed to incorporate this emerging knowledge into health protective practices.

There are now well-described instances of human *in utero* exposures that have produced significant increases in later-life susceptibility to disease. Best known are the studies of the Dutch famine (Painter et al. 2005). During the winter of 1944–1945, toward the end of World War II, the population in German-occupied western Holland had only very limited food available, with an average daily intake of < 1,000 calories for several months. Children born to women who were pregnant during this famine were small for gestational age (SGA). Later in life, this *in utero*–deprived cohort developed an increased incidence of various adult-onset diseases, including obesity, diabetes, cardiovascular disease, and renal dysfunction. In addition, the children born to members of this *in utero*–deprived cohort were also SGA, indicating a passage of this predilection through generations (Painter et al. 2008). Another example of the later-life consequences of an early-life chemical exposure is *in utero* and early childhood exposure to arsenic-contaminated drinking water in Chile. Beginning in 1958, with the development of a new water supply, the population of a large town in the Antofagasta region of northern Chile was exposed to very high levels of arsenic [~ 800 ppb in drinking water; the U.S. Environmental Protection Agency (EPA) maximum contaminant level is 10 ppb], an exposure that abruptly ended with the institution of water filtration in 1970 (Dauphine et al. 2011; Smith et al. 2006; Yuan et al. 2007). The cohort of individuals exposed to arsenic early in life was later found to have significant deficiencies in lung function and increases in cardiovascular mortality compared with a nonexposed control group (Dauphine et al. 2011; Smith et al. 2006; Yuan et al. 2007).

The potential scope of the problem is illustrated by an example from the recent economics literature, where evidence of effects of the 1918 Spanish flu pandemic was seen in the economic performance and achievements of its victims. Men born to U.S. mothers who contracted the flu while pregnant had reduced educational attainment, increased rates of physical disabilities, lower socioeconomic status, 5–9% overall lower income, and approximately 30% greater welfare payments (Almond 2006). In a Brazilian cohort born during and soon after this same flu pandemic, children of flu-exposed mothers were less likely to be literate, to have graduated college, to be employed, or to ever have had formal employment (Nelson 2010). Although not every association seen in the U.S. cohort was observed in the Brazilian counterpart—and despite the fact that no aggregate economic impact number has been estimated—the results between the two studies were impressively concordant. The end result of this body of work is the powerful indication that early-life exposures have a strong, significant, and long-lasting effect on later-life function and disease in this circumstance.

Here we discuss examples of human exposures to adverse intrauterine environments that underscore the dramatic biological consequences of interference with normal development. Human data provide strong biological plausibility connecting early-life exposures to later-life disease and raise the important question of the underlying molecular mechanisms responsible for these long-lasting effects. Because alterations in DNA sequence per se do not explain the later-life effects of these exposures, epigenetic mechanisms have been invoked. To study these epigenetic mechanisms in detail has required the development of appropriate animal models such as the agouti mouse (Dolinoy et al. 2007). To date, this emerging knowledge has not been incorporated into risk assessment processes or regulatory practice. Indeed, significant scientific and conceptual barriers must still be overcome as health protective measures are developed for early-life exposures that induce molecular effects resulting in later-life disease. In this review, we explore the scientific basis for these latent effects and discuss the risk assessment context, forming the basis for the development of a research agenda designed to generate the knowledge base necessary for understanding and appropriately regulating early-life exposures.

## Results

*Early-life exposures, later-life effects, and epigenetic mechanisms.* In humans, early insults are associated with later-life liabilities, including prematurity, low birth weight, maternal infection during pregnancy, toxic exposures, and malnutrition. Premature birth (< 37 completed weeks gestation) is an important early-life event of increasing incidence (Martin 2011). Decreasing age at birth has been associated with increased odds of high systolic blood pressure in a population-based cohort study of young adult men (Johansson et al. 2005), and premature birth before 35 weeks gestational age predicted the development of diabetes in both adult men and women (Kajantie et al. 2010; Pilgaard et al. 2010).

Maternal infection during pregnancy has been associated with neuropsychiatric disorders such as autism and schizophrenia (Brown and Patterson 2011; Meyer et al. 2011; Patterson 2011). Both human and animal studies support this association, with suggested mechanisms ranging from altered hippocampal neurotransmitter signaling to persistent chronic inflammation (Baharnoori et al. 2010; Buehler 2011; Meyer et al. 2008; Moreno et al. 2011).

Numerous early-life toxic exposures have been linked to later-life health effects, with particularly strong evidence regarding maternal smoking during pregnancy being predictive of impaired fertility, obesity, hypertension, and neurobehavioral deficits (Bruin et al. 2010; Gustafsson and Kallen 2011; Heinonen et al. 2011; Simonetti et al. 2011; Thiering et al. 2011). Animal studies suggest that nicotine alone may be enough to elicit the long-term consequences of maternal smoking on progeny (Bruin et al. 2010) and that prenatal and perinatal toxicant exposures can result in latent effects, including elevated blood pressure, insulin resistance, and obesity (Fraites et al. 2009; Lassiter et al. 2008; Leasure et al. 2008).

**Figure 1:**
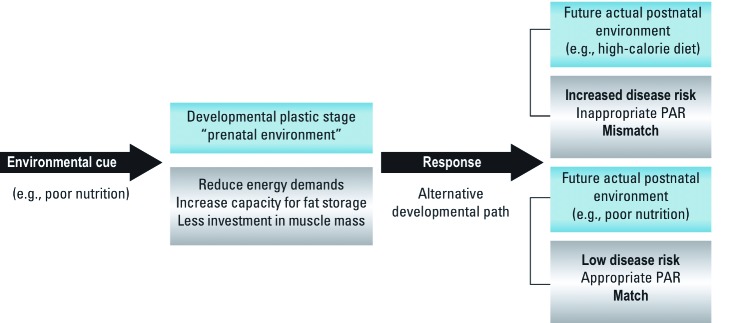
The combination of maternal nutrition (i.e., *in utero* nutrition) and postnatal nutrition can be adaptive or maladaptive, leading to increased or decreased disease risk later in life. During development, an organism responds to an environmental stimulus by shifting its developmental path to a phenotype that confers a survival or reproductive advantage in postnatal life; this process of programmed adaptation is called predictive adaptive response (PAR).

Malnutrition is an example of an environmental stressor that invokes a predictive adaptive response in the developing organism (Hanson et al. 2011). The fetus appears to use the *in utero* environment to predict and prepare for the postnatal environment. That is, an organism alters its developmental path to produce a phenotype that gives it a survival or reproductive advantage in postnatal life (Figure 1).

Fetal malnutrition can be defined as either overnutrition or undernutrition, or as a deficiency of specific nutrients. Studies of the Dutch famine of 1944–1945 have implicated maternal undernutrition in the pathogenesis of multiple diseases, including coronary heart disease, hypertension, obesity, insulin resistance, and schizophrenia (de Rooij et al. 2006; Painter et al. 2005; Ravelli et al. 1976, 1998; Roseboom et al. 2000; Susser et al. 1996). Fetal nutrition is also affected by placental dysfunction and uteroplacental insufficiency. In developed countries, the diseases associated with placental insufficiency, such as maternal smoking and pregnancy-induced hypertension (e.g., preeclampsia), are the most common causes of fetal undernutrition (Bergmann et al. 2008; Henriksen and Clausen 2002). Preeclampsia, for example, affects up to 3 million people in the United Kingdom and 15 million people in the United States (Davis et al. 2012). Moreover, the incidence of diseases such as preeclampsia is increasing in developing countries (Lopez-Jaramillo et al. 2005). Fetal undernutrition leads to intrauterine growth restriction (IUGR; the failure of the fetus to reach its genetic growth potential due to a pathological event). IUGR in humans predicts adult disease, including hypertension, diabetes, obesity, cardiovascular disease, respiratory dysfunction, and neurocognitive disease (Joss-Moore and Lane 2009; Lahti et al. 2006; Varvarigou 2010). The relationship between IUGR and adult disease is often studied using measures such as the ponderal index (birth weight × 100/crown–heel length^3^). The ponderal index reflects fetal undernutrition because, when presented with limited nutrition, the fetus will maintain body length but not body weight (asymmetric growth restriction or brain sparing) (Bartha et al. 1998; Fok et al. 2009; Patterson and Pouliot 1987). A reduced ponderal index has been associated with multiple diseases, including insulin resistance, obesity, behavioral symptoms of attention deficit hyperactivity disorder, coronary heart disease, and hypertension (Fan et al. 2010; Jarvelin et al. 2004; Lahti et al. 2006; Lithell et al. 1996; Loaiza et al. 2011; Walther 1988). Other measures of fetal malnutrition in the newborn that have been predictive of adult disease include placental efficiency (fetal weight/placental weight), placental morphometry, and combinations of maternal and placental size (Eriksson et al. 2011; Hemachandra et al. 2006).

IUGR in animals similarly predicts adult morbidities such as hypertension, obesity, and insulin resistance (Baserga et al. 2007; Desai et al. 2005, 2007; Joss-Moore et al. 2010b; Simmons et al. 2001; Tsirka et al. 2001). Common animal models of fetal malnutrition include placental insufficiency, food restriction, protein restriction, micronutrient deficiency, and glucocorticoid exposure. Although the molecular pathogenesis may differ, these models give rise to a similar profile of adult diseases. Both offspring sex and the timing of exposure influence the later-life consequences of exposure (Fu et al. 2009; Ke et al. 2006).

**Figure 2:**
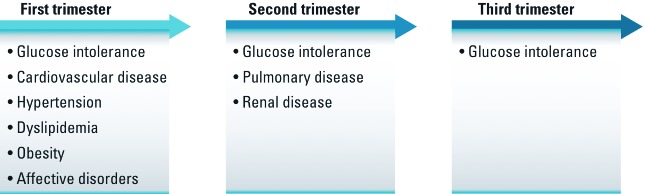
Studies of the Dutch famine birth cohort underscore the importance of timing in developmental processes. The timing of in utero nutritional deprivation is associated with different later-life disease outcomes (Roseboom et al. 2001, 2006).

Timing and intergenerational effects of exposures. The Dutch famine cohort illustrates the critical effect of timing of exposures in humans (Figure 2). Individuals who were *in utero* early in gestation during the famine suffer from an increased incidence of coronary heart disease, hypertension, dyslipidemia, and obesity, whereas those who were *in utero* midgestation suffer from an increased incidence of obstructive airway disease and impaired glucose tolerance (Roseboom et al. 2001, 2006).

In an examination of the impact of exposures across generations using the Dutch famine cohort, Painter et al. (2008) found evidence that progeny (F_2_) of women (F_1_) born during the famine suffered from increased neonatal adiposity and poor adult health, including neurological disorders, and respiratory and autoimmune conditions. Animal studies also provide support for the occurrence of intergenerational consequences of exposure. F_1_ female rats, hypertensive as a result of prenatal malnutrition, transmit the predisposition toward hypertension and endothelial dysfunction to their F_2_ progeny (Torrens et al. 2008). F_1_ male rats exposed to the endocrine disruptor vinclozolin *in utero* may also pass on a number of disease states involving prostate, kidney, immune system, and metabolism (hypercholesterolemia) to the F_4_ generation (Anway et al. 2006).

**Figure 3:**
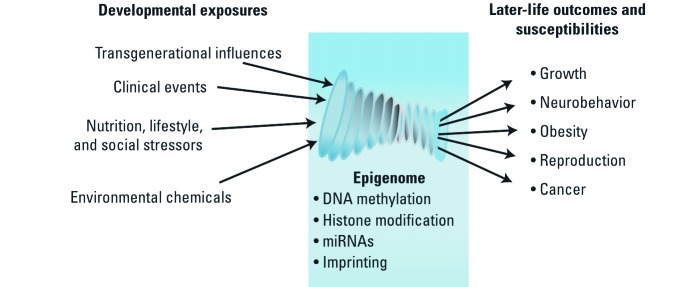
Developmental exposures are focused through the lens of epigenetic mechanisms to influence later-life disease outcomes and susceptibilities.

Epigenetic mechanisms. The underlying mechanism responsible for these myriad effects of exposures is, at least in part, epigenetic (Figure 3). Epigenetics forms the basis of how eukaryotes regulate gene expression. Epigenetic modifications direct access of the transcriptional machinery and cofactors to regulatory regions of the gene, modulating transcriptional initiation, elongation, and termination. Studies in multiple model systems demonstrate that epigenetic modifications are important along the entire gene, including untranslated regions (Einstein et al. 2010; Fu et al. 2006, 2009).

Epigenetic modifications operate as an “on/off switch” to regulate gene expression, as with imprinting, or as a rheostat control to increase or decrease expression. Epigenetic modifications that function as a rheostat are often driven by environmental conditions, particularly those that are extreme, and are thought to function as a cellular memory of previous environmental conditions (Hanson et al. 2011), so that future environments can be physiologically anticipated. In this context, epigenetic modifications may represent accessible molecular markers of early-life events that may be more stable than direct measures of gene expression. The epigenetic modifications most often studied include the histone code, DNA CpG methylation, and microRNA (miRNA) expression levels.

The histone code contains the most capacity for epigenetic modification (Ruthenburg et al. 2007). Each cell contains approximately 3 × 10^8^ nucleosomes, each consisting of histone core proteins that can be differentially acetylated, methylated, or phosphorylated. Presumably, cells and tissues adjust their histone codes during development in response to ambient conditions to fine tune the regulation of gene expression in anticipation of the future extrauterine environment.

DNA methylation occurs on cytosines in CpG sequences and is associated with specific early-life events, including maternal malnutrition, *in vitro* fertilization, transplacental exposures, and IUGR (Gomes et al. 2009; Heijmans et al. 2008; Katari et al. 2009; Perera et al. 2009; Steegers-Theunissen et al. 2009; Tobi et al. 2009). Although many different early-life events lead to similar adult phenotypes, the epigenetic modifications that occur in these exposures may be different; in other words, the end result may be the same, but the pathways giving rise to that result may be different.

miRNAs are a class of small RNAs 21–25 nucleotides in length that act as posttranscriptional regulators of gene expression (Du and Zamore 2007). These small RNAs bind to the 3´-untranslated regions of target mRNA transcripts and disrupt the translational machinery or lead to transcript degradation, depending on the level of sequence complimentarity. Humans have approximately 1,000 miRNAs, each of which can interact with a family of target RNAs, creating a regulatory mechanism with the potential to modulate about 60% of the protein-coding genes (Sayed and Abdellatif 2011).

Placenta. The placenta, a readily accessible human tissue that may reflect the fetal environment, could prove to be a very useful tool for understanding the mechanisms underpinning the developmental origins of disease; it could also serve as a tissue source for biomarkers to predict later disease risk. The placenta grows and develops throughout gestation in a dynamic, highly orchestrated manner (Myatt and Roberts 2006). Placental vascular development is important for the transfer of flow-limited substrates and for fetal cardiovascular loading and heart fitness. Placental growth is under the control of imprinted genes that may up- or down-regulate growth depending on the parent of origin. Placental 11-β-hydroxysteroid dehydrogenase-2 (*11*β*HSD2*) activity is developmentally regulated, responds to nutrients and oxygen levels, and regulates fetal exposure to maternal cortisol (Li et al. 2011). Oxidative stress is increasingly seen as a regulator of placental and fetal growth and development (Myatt 2010). Pregnancies complicated by obesity, preeclampsia, and IUGR are associated with increased placental oxidative and nitrative stress, leading to covalent modifications of placental proteins based on observational studies in humans (Myatt 2010).

The placenta has a distinct DNA methylation profile that changes throughout gestation in response to environmental cues (Chu et al. 2011; Novakovic et al. 2011). Placental epigenetic biomarkers have emerged as an active and informative area for the identification of early-life indicators of later-life disease (Maccani and Marsit 2009). Infant growth restriction has been associated with distinct patterns of placental DNA methylation (Banister et al. 2011). Placentas from large-for-gestational-age newborns had differential methylation of the glucocorticoid receptor (*GR*) gene (Filiberto et al. 2011). Maternal smoking has been associated with down-regulation of the placental miRNAs *miR-16*, *miR-21*, and *miR-126a* (Maccani et al. 2010), and reduced expression of *miR-16* and *miR-21* has been associated with SGA newborns (Maccani et al. 2011). Therefore, the placenta is a source of integrated molecular information about the developmental life history of the fetus and its environmental interactions.

In utero *and postnatal epigenetic modifications that predict end points such as obesity, insulin resistance, and hypertension.* In mice, differences in micronutrient intake during pregnancy induced differences in the coat color of offspring due to hypomethylation of the 5´ end of the agouti gene (Wolff et al. 1998), whereas a protein-restricted diet during pregnancy led to hypomethylation of promoter regions of metabolically important regulators—the *GR* and peroxisome proliferator-activated receptor (*PPAR*) α genes (Lillycrop et al. 2007). In this rodent model, hypomethylation of *GR* and *PPAR*α was accompanied by an increase in their expression and that of their target genes, *PEPCK* (phosphoenolpyruvate carboxykinase) and *AOX* (acyl CoA oxidase), and the metabolic processes that they control, namely, glucogeneogenesis and β-oxidation. Altered methylation status of the liver *PPAR*α promoter in juvenile offspring was due to hypomethylation of four specific CpG dinucleotides, two of which predicted the level of the mRNA transcript and persisted into adulthood (Lillycrop et al. 2008). The differentially methylated CpGs corresponded to transcription factor binding sites, which suggests that changes in the epigenetic regulation of genes established during development will induce altered transcription in response to specific stimuli and modify the capacity of the tissue to respond to metabolic challenge. Other animal studies have shown that folic acid supplementation (Wolff et al. 1998), neonatal overfeeding (Plagemann et al. 2009), constrained intrauterine blood supply (Pham et al. 2003), and maternal behavior (Weaver et al. 2004) alter the epigenetic regulation of genes in the offspring and that these changes are associated with an altered phenotype.

Hypomethylation of the imprinted *IGF2* (insulin-like growth factor 2) gene has been observed in genomic DNA isolated from whole blood from 60 individuals who were exposed periconceptually to famine *in utero* during the Dutch famine compared with their unexposed same-sex siblings (Heijmans et al. 2008). In two independent cohorts, the methylation status of a single CpG site in the promoter region of the retinoid X receptor α (*RXR*α) in the umbilical cord was positively associated with childhood adiposity in both boys and girls, such that *RXR*α promoter methylation explained more than one-fifth of the variance in childhood fat mass (Godfrey et al. 2011). These human studies indicate that epigenetic marks may allow identification of individuals at increased risk of chronic disease in later life before the onset of clinical disease, thus facilitating targeted intervention strategies.

PPARγ and obesity. Rates of obesity have increased in infants, young children, and adolescents (Koebnick et al. 2010; McCormick et al. 2010; Taveras et al. 2009), suggesting that obesity is being programmed prenatally or in early childhood. Growing evidence supports a contribution of endocrine-disrupting chemicals (EDCs) in the obesity epidemic, and mechanisms are being revealed for at least a few EDCs (Janesick and Blumberg 2011a). Obesogens are chemicals that promote obesity by increasing the number of fat cells (and fat storage into existing fat cells), by changing the amount of calories burned at rest, by altering energy balance to favor storage of calories, and by altering the mechanisms through which the body regulates appetite and satiety (reviewed by Janesick and Blumberg 2011a). PPARγ plays an important role in nearly all aspects of adipocyte biology and is thought to be the master regulator of adipogenesis (Evans et al. 2004; Tontonoz and Spiegelman 2008). Activation of PPARγ2 in preadipocytes increases their differentiation into adipocytes, and PPARγ is required for adipocyte differentiation *in vitro* and *in vivo* (Rosen et al. 1999). The ligand-binding pocket of PPARγ is large and considered to be promiscuous (Maloney and Waxman 1999). A number of chemicals act as PPARγ ligands, many of which are obesogenic (Janesick and Blumberg 2011b).

One obesogen for which a mechanism of action is known is the organotin tribuytltin (TBT) (Grun et al. 2006). In mice, a single prenatal exposure to TBT during gestation resulted in premature accumulation of fat in adipose tissues and increased size of the fat depot relative to overall body mass (Grun et al. 2006). In mouse pups born to TBT-treated mothers, the liver, testis, mammary gland, and inguinal adipose tissue, which normally do not store lipids before feeding commences, all had stored fat at birth (Grun et al. 2006). TBT has a nanomolar affinity for both RXR and PPARγ, activates PPARγ–RXR heterodimer binding to DNA, and directly regulates transcription of its target genes (Grun et al. 2006; Kanayama et al. 2005; Tontonoz and Spiegelman 2008).

Mature adipocytes are generated from multipotent stromal cells (MSCs) found in almost all fetal and adult tissues (da Silva Meirelles et al. 2006). MSCs can differentiate into bone or adipose tissue, a balance mediated by PPARγ (reviewed by Takada et al. 2009). Intriguingly, exposure to the environmental obesogen TBT or the pharmaceutical obesogen rosiglitazone have been reported to induce the differentiation of MSCs into adipocytes at the expense of bone via PPARγ activation (Kirchner et al. 2010). Moreover, pregnant dams treated with a single dose of TBT or rosiglitazone produced pups with MSCs that *in vitro* differentiated into adipocytes about twice as frequently as did MSCs from controls (Kirchner et al. 2010). Thiazolidinedione antidiabetic drugs such as rosiglitazone are potent activators of PPARγ (Lehmann et al. 1995) and are known to increase weight and fat cell number in humans (Shadid and Jensen 2003).

MSCs derived from mice exposed to TBT *in utero* have exhibited alterations in the methylation status of the CpG islands of adipogenic genes such as *AP2* and *PPAR*γ. This altered methylation was associated with an increased number of preadipocytes in the MSC compartment and an increased frequency with which MSCs differentiate into adipocytes upon adipogenic stimulation (Kirchner et al. 2010). Understanding how adipocyte number is programmed at the genomic level will be of critical importance in understanding how the set point for adipocyte number is modified by chemicals, dietary factors, or the intrauterine environment.

In utero *and postnatal indicators that predict diseases caused by arsenic exposure*. Early-life exposure to inorganic arsenic produces a wide range of malignant and nonmalignant diseases in humans. Exposure to arsenic from naturally contaminated drinking water affects roughly 140 million people worldwide (Pilsner et al. 2009). For example, in Bangladesh, where exposure began in the early 1970s, there is a generation of women and men who have been exposed to arsenic for their entire lives. The placenta is not a barrier to arsenic; thus, children are born with blood concentrations of arsenic and its toxic metabolites similar to those present in their mothers (Hall et al. 2007). Increased lung cancer and bronchiectasis have been reported in young Chilean men and women who were exposed to arsenic only during prenatal and early postnatal life (Smith et al. 2006). Long-term follow-up of a cohort of thousands of infants exposed to arsenic-contaminated milk powder in 1955 in Japan suggests increased rates of leukemia and skin, liver, and pancreatic cancers (Yorifuji et al. 2010). In Thailand, among babies of mothers who experienced varying degrees of arsenic exposure, gene expression profiles were indicative of the activation of molecular networks associated with inflammation, apoptosis, stress, and metal exposure (Fry et al. 2007). In arsenic-exposed adults, DNA hypomethylation has been associated with the subsequent risk of developing arsenic-induced skin lesions (Pilsner et al. 2009). Collectively, this body of work in human populations strongly suggests that developmental exposure to arsenic may induce alterations in fetal cellular functioning that may have major public health consequences, including cancer, in later life (Ren et al. 2011; Tokar et al. 2011).

Arsenic as a transplacental carcinogen in mice. Comparing exposure levels between mice and humans is complicated by differences in metabolism and excretion (Carter et al. 2003; States et al. 2011). A transplacental model has been developed in which mice receive inorganic arsenic in the drinking water only during pregnancy. In this model arsenic acts as either a complete carcinogen or enhances cancer response to other agents in offspring as adults (Tokar et al. 2011). Transplacental exposure to arsenic in mice has been reported to produce tumors or stimulate response to other agents in various target tissues, including sites concordant with human targets of arsenic. Developing theory posits that cancer often is a stem cell (SC)-based disease. SCs are particularly active during the perinatal period. In fact, Tokar et al. (2011) reported that mouse skin carcinomas stimulated in adult offspring by prenatal arsenic exposure were remarkably enriched in cancer SCs. These authors reported that during malignant transformation by arsenic *in vitro*, a survival selection of SCs occurred, which resulted in an overabundance of cancer SCs (compared with other carcinogens) as cancer phenotype was acquired (Tokar et al. 2011). Thus, it appears that arsenic targets long-lived SC populations—perhaps through epigenetic modifications—to cause or to facilitate carcinogenic events during adulthood as a possible mechanism of the developmental basis of adult disease (Tokar et al. 2011).

Arsenic exposure and cardiovascular disease in mice. Myocardial infarction in infants exposed to high levels of arsenic during fetal life (Rosenberg 1974) was the first indication that *in utero* arsenic exposure could cause advanced atherosclerosis. In the apoplipoprotein E–knockout mouse, a commonly used model for human atherosclerosis, both *in utero* (Srivastava et al. 2007) and postweaning (Srivastava et al. 2009) arsenic exposures accelerate and exacerbate atherosclerosis. The postweaning studies have shown a clear linear dose response, further confirming the cardiovascular risk from arsenic exposure. These animal model results are consistent with early epidemiologic findings in Taiwan (Chen et al. 1996) and recent findings in Bangladesh (Chen et al. 2011) that show an arsenic dose response related to mortality from cardiovascular disease.

## Discussion

When we consider all of the human and animal evidence together (Table 1), many questions and research directions arise as to how we should develop and improve upon strategies to identify the individuals in whom early-life events predict adult diseases:

**Table 1:** Examples of *in utero* exposures that result in adverse health outcomes later in life.

Species and health outcome, phenotype, or condition	Mechanistic-, biomarker-, or epigenetic-related finding
Diet, maternal behavior, or fetal growth		
Human
Maternal diet during Dutch famine (1944–1945): obesity, diabetes, insulin resistance, coronary heart disease, hypertension, renal dysfunction, schizophrenia, and SGA in the birth cohort (de Rooij et al. 2006; Painter et al. 2005; Ravelli et al. 1976, 1998; Roseboom et al. 2000; Susser et al. 1996); association with health outcome is dependent on trimester of exposure	Hypomethylation of IGF2 gene (Heijmans et al. 2008)
IUGR: hypertension, diabetes, obesity, cardiovascular disease, respiratory dysfunction, neurological disorders (Joss-Moore and Lane 2009; Lahti et al. 2006; Varvarigou 2010; Walther 1988)	Putative biomarkers of metabolic syndrome: myoinositol, sarcosine, creatine, creatinine (Dessi et al. 2011)
Reduced ponderal index: insulin resistance, obesity, behavioral symptoms of attention deficit hyperactivity disorder, coronary heart disease, hypertension (Fan et al. 2010; Jarvelin et al. 2004; Lahti et al. 2006; Lithell et al. 1996; Loaiza et al. 2011; Walther 1988)	Putative biomarkers: maternal body size, placental morphometry (Eriksson et al. 2011)
Premature birth: high blood pressure (males) and diabetes (Johansson et al. 2005; Kajantie et al. 2010; Martin 2011; Pilgaard et al. 2010)	Markers that vary with gestational age: hippurate, tryptophan, phenylalanine, malate, tyrosine, hydroxybutyrate (Atzori et al. 2011)
Animal
Protein- or calorie-restricted diet: increased blood pressure, impaired glucose homeostasis, decreased insulin sensitivity (Armitage et al. 2004)	Hypomethylation of promoter regions of GR and PPARα (Lillycrop et al. 2007) Altered hepatic transcriptome (Lillycrop et al. 2010)
Uteroplacental insufficiency: IUGR	Increased renal apoptosis Altered p53 DNA CpG methylation (Pham et al. 2003)
Prenatal or neonatal overfeeding: rapid early weight gain, metabolic syndrome phenotype (obesity, hyperleptinemia, hyperglycemia, hyperinsulinemia, increased insulin/glucose ratio) (Plagemann et al. 2009)	Hypermethylation of proopiomelanocortin promoter (Plagemann et al. 2009)
Methyl-supplemented diet: shifting of coat color and adiposity in the Avy mouse (Waterland 2006)	Decreased expression of Avy epiallele and hypermethylation of the agouti Avy metastable epiallele
High or low maternal licking and grooming of pups: female offspring have same high or low grooming behavior as their mothers (Weaver et al. 2004)	Differences in GR expression and DNA methylation of GR gene promoter in hippocampus (Weaver et al. 2004)
Maternal illness during pregnancy		
Human
Infection: autism and schizophrenia (Brown and Patterson 2011; Meyer et al. 2011; Patterson 2011)
Spanish flu pandemic, United States and Brazil: reduced educational attainment, increased rates of physical disabilities, lower socioeconomic status in males (Almond 2006; Nelson 2010)
Chemical exposure during pregnancy or infancy		
Human
Maternal smoking: impaired fertility, obesity, hypertension, neurobehavioral deficits (Gustafsson and Kallen 2011; Heinonen et al. 2011; Simonetti et al. 2011; Thiering et al. 2011)	Biomarkers of exposure: cord blood, meconium, saliva nicotine and cotinine
Arsenic in northern Chile, 1958–1970: lung cancer, impaired lung function, myocardial infarction in infants, cardiovascular mortality in adults (Dauphine et al. 2011; Rosenberg 1974; Smith et al. 2006; Yuan et al. 2007) Japan, 1955 (contaminated milk powder): leukemia, skin, liver, and pancreatic cancers in adults exposed as neonates (Yorifuji et al. 2010)	Gene expression profiles changes in Thai infants were indicative of the activation of molecular networks associated with inflammation, apoptosis, stress, and metal exposure (Fry et al. 2007)
Perfluorooctanoic acid in Denmark: positive association between maternal serum PFOA at 30 weeks gestation and overweight/obesity, serum insulin, and leptin in females at 20 years of age; negative association with adiponectin
Animal
Arsenic: a transplacental carcinogen in mice; enhances cancer response to other agents in adult animals (Tokar et al. 2011)	Treatment of stem cells during malignant transformation by arsenic in utero results in an overabundance of cancer SCs as cancer phenotype is acquired (Tokar et al. 2011)
Perfluorooctanoic acid: increased body weight in midadulthood, increased serum leptin and insulin (Hines et al. 2009)
Diethylstilbestrol: female reproductive tract cancer and malformations; male reproductive tract anomalies, increased risk of breast cancer at > 40 years of age (Adami et al. 2012; Rubin 2007)
Diethylstilbestrol: reproductive tract anomalies and obesity in females (Newbold et al. 2009)	Estrogen receptor mediated
Dexamethasone: lower birth weight, hypertension, hyperglycemia, insulin resistance, enhanced stress response, obesity (variable), premature differentiation of organs and tissues (Cleasby et al. 2003; O’Regan et al. 2008; Seckl et al. 2000; Seckl and Meaney 2004)	Altered GR expression, decreased placental 11β-HSD2 Altered methylation of GR promoters (Weaver et al. 2004)
TBT: fat accumulation in adipose tissue in F1 mice (Grun et al. 2006); MSCs from F1 pups differentiate into adipocytes about twice as frequently in culture as MSCs from controls (Kirchner et al. 2010)	Nanomolar affinity for RXR and PPARγ, activates PPARγ–RXR heterodimer binding to DNA and directly regulates transcription of its target genes (Grun and Blumberg 2006; Kanayama et al. 2005; Tontonoz and Spiegelman 2008) MSCs derived from mice exhibited alterations in the methylation status of the CpG islands of adipogenic genes such as AP2 and PPARγ (Kirchner et al. 2010)

Can epigenetic biomarkers be used to identify mechanisms, so we can treat the cause and not the symptom?
How do we assess the information stored in the histone code?
Can epigenetic biomarkers be used as integrative measures of mixed exposures?
How do we prioritize the investigation of different epigenetic mechanisms (DNA methylation, histone modifications, miRNAs) involving different sites within the epigenome?
What are the most critical developmental time points for producing a later-life effect of an exposure?
What are nonepigenetic mechanisms by which early-life events alter adult disease risk, and are there early markers for these?
How relevant is sex and tissue specificity?

Development of improved molecular and computational approaches that can generate and sift through exponentially increasing amounts of data is an overarching research priority. Just as important, understanding the epigenetic and nonepigenetic mechanisms that underlie the relationship between early-life events and adult disease risk will be essential in terms of *a*) interpreting the data, *b*) prioritizing the findings, and *c*) designing specific interventions that treat causes—both environmental and physiologic—and not only markers of adverse consequences of early-life events.

The pleiotropic effects of arsenic exposure on human health and in animal models illustrate an important area for further research. Animal studies suggest that the varied disease outcomes resulting from similar exposures are dependent on the disease predilection of the animal. Thus, *in utero* arsenic exposure induces cancer in adulthood in cancer-susceptible mouse strains and cardiovascular disease in atherosclerosis-prone strains. These observations suggest that the particular disease manifestation of arsenic exposure in humans may be dependent on the genetic predisposition of the individual as well as the life-stage timing of exposure, and that arsenic exposure may accelerate an underlying predilection to pathology. For example, a single nucleotide polymorphism near the gene for arsenic methyltransferase has been associated with an increased risk for arsenic-induced skin lesions (Pierce et al. 2012). It is not clear whether cessation of arsenic exposure can reduce the disease incidence, but emerging human data indicate that short, high-level arsenic exposure in early life is linked to cancer (Yorifuji et al. 2010), again emphasizing life-stage timing of exposure. Future research focusing on plasma biomarkers of disease in animal models and translating these to human populations will be of great benefit in identifying disease risk and in developing potential intervention strategies. In addition, large-scale genetic association studies in arsenic-exposed humans may provide valuable clues about disease susceptibility in both arsenic-exposed and unexposed populations.

*How do we define the scope of this problem?* We envision three distinct research strategies that can be used to define the scope and importance of early-life exposures predisposing later-life disease. The first approach would identify changes in known epigenetic targets already shown to be responsive following exposures of interest using high throughput *in vitro* systems and regulating on the result. However, this approach may be premature and too narrow scientifically because of our limited understanding of the underlying biology. A second approach would add a number of preliminary end points (such as epigenetic modifications) and pathway measures (such as DNA methylation capability) onto the very early part of a 2-year rodent bioassay beginning with an *in utero* exposure; early biomarkers could then be associated with later-life disease. This approach would produce large amounts of data, but at great cost in time and resources. An intermediate approach would be to assemble a comprehensive list of possible end points and pathways that might plausibly link exposure to a response (to the degree that this is known now) and then test a number of known “bad actors” (and their inactive congeners) to see which combinations of pathways and end points can separate the known actives from the known inactives. This approach would be limited to pathways that are currently known, but an advantage would be that it would generate some survey-scale data relatively quickly. Undoubtedly, all three of these research approaches will be used going forward to provide risk assessors with sufficiently robust data to begin the risk characterization of the relationship between early-life exposure and later-life disease.

*How do we incorporate this emerging knowledge into risk assessment practices and health protective policies?* The emerging science described here may be instructive for risk assessment and public health decision making. To be useful in these processes, science must address questions of interest to risk managers, including

What adverse effects will result from exposure (hazard identification)?
At what level of exposure will these effects occur (exposure/dose–response relationships)?
How certain are we of these effects (risk characterization)?
These questions are discussed below, exploring the extent to which early-life indicators of later-life disease may inform risk assessment.

Hazard identification. Both animal and human evidence now support a causative relationship between early-life exposures and a wide variety of later-life diseases (Schug et al. 2011). There is also an emerging understanding of the underlying mechanisms through which this can occur. The examples presented above focus primarily, but not exclusively, on epigenetic mechanisms and include exposures to chemicals, malnutrition, and other environmental stressors. As stated by Burdge and Lillycrop (2010),

[the] process that underlies induction of differential risk of disease by variation in the prenatal environment reflects environmental cues acting through developmental plasticity, which generate a range of genotypes from a single genome …. Recent findings show that altered epigenetic regulation of specific genes is central to the process by which different phenotypes are generated and hence differential risk of disease.

Chemicals and other environmental agents appear to have the ability to miscue the developing organism, resulting in maladaptation associated with increased disease (Janesick and Blumberg 2011a; Joss-Moore et al. 2010a). Alterations in DNA methylation, chromatin remodeling, and miRNA expression can modify SC fate and produce persistent changes in gene expression, resulting in downstream effects on cell, tissue, and organism functions in later life. These perturbed epigenetic targets can be used as early indicators of adverse health effects. Confidence in these early events as indicators is based on a systems biology level of understanding, compared with alterations in single isolated events. For specific, well-developed examples, such as *IGF2* hypomethylation in Dutch famine victims and increased methylation of *RXR*α at birth associated with childhood adiposity, the case for causal links between these upstream molecular events and later-life effects is compelling. In certain situations, evidence may be sufficient to describe the upstream event or indicator itself as an adverse effect.

Exposure/dose–response relationships. For arsenic and obesogens, quantitative relationships between exposure or dose response and various indicators are evident, thus establishing their role as causal biomarkers of exposures. Currently, the quantitative relationships between these indicators and risks of disease must be experimentally or observationally determined. Given the complex and multifactorial nature of disease risks, it is not yet possible to predict quantitatively the risk of disease from the indicator exposure response or dose response alone. Complicating factors are *a*) the specific disease is contingent on interactions with the environment throughout the life course; *b*) the timeframe of environmental exposure, relative to the stage of *in utero* development, can influence the type of disease observed in later life; and *c*) identification of appropriate indicators from among the variety of associative and secondary changes occurring within the body is clearly complicated and difficult. Fortunately, this field is rapidly developing, and further advances will be aided by the increasing mechanistic understanding of human variability in response and the role of complex environmental exposures in disease risks.

Risk characterization. For the examples discussed above, evidence for a causal relationship between specific early indicators and adverse outcomes is provided by experimental evidence in animals and supported by observational human data. Taken together, the evidence for a causal relationship between early-life exposures, specific early indicators, and later-life disease is consistent, coherent across various types of studies, and biologically plausible. These examples illustrate the types of data and approaches that can inform risk assessment, as well as the substantial challenges that remain for general use of these types of data in risk assessment.

## Conclusion

The examples presented here of early-life exposures that result in later-life disease provide both proof of concept and insights into the value of specific information. Although insufficient data are available on a substantial number of chemicals or end points for widespread application in current risk assessments, this new knowledge paves the way to a deeper understanding of the underlying biology and evaluation of potential public health risks. To further the use of these types of data in future risk assessments, additional basic biological research aimed at increasing knowledge of inherent epigenetic and gene regulatory structures of targeted genomic regions and their roles in disease must be supported. These new approaches have significant potential in generating novel causal biomarkers of exposure and of increasing understanding of susceptibility and responses to complex environmental exposures, thus serving as sophisticated indicators of potential risks.
